# Development of a Bead-Based Multiplex Fluorescent Immunoassay to Detect Antibodies against Maedi-Visna Virus in Sheep

**DOI:** 10.3390/ani14101442

**Published:** 2024-05-12

**Authors:** Anniken Jerre Borge, Barbara Colitti, Sergio Rosati, Anne B. Nordstoga, Britt Gjerset, Kristin Udjus, Chiara Nogarol, Stalin Chellappa, Ingunn Anita Samdal, Kari Lybeck

**Affiliations:** 1 Norwegian Veterinary Institute, P.O. Box 64, 1431 Ås, Norway; anne.nordstoga@vetinst.no (A.B.N.); britt.gjerset@vetinst.no (B.G.); kristin.udjus@vetinst.no (K.U.); stalin.chellappa.gunasekaran@vetinst.no (S.C.); ingunn.samdal@vetinst.no (I.A.S.); kari.lybeck@vetinst.no (K.L.); 2 Department of Veterinary Science, University of Turin, Largo P. Braccini 2, 10095 Grugliasco, TO, Italy; barbara.colitti@unito.it (B.C.); sergio.rosati@unito.it (S.R.); 3 In3diagnostic s.r.l., Largo P. Braccini 2, 10095 Grugliasco, TO, Italy

**Keywords:** small ruminant lentivirus, SRLV, MVV, ovine, diagnostic test, luminex immunoassay

## Abstract

**Simple Summary:**

Serological tests that detect antibodies, especially indirect ELISAs, are the most common method to diagnose Maedi-visna virus (MVV) infections in small ruminants. However, due to the high genetic heterogeneity of the virus, commercial tests might not effectively detect infected animals in all populations. Bead-based multiplex immunoassays can detect multiple analytes simultaneously, thus enabling the detection of antibodies against several MVV antigens in the same assay. In Norway, there is a national aim to eliminate MVV from the sheep population and improving the serological test performance would strengthen the surveillance of the disease. In this study, we developed a bead-based multiplex immunoassay to detect antibodies against viral epitopes in MVV-infected sheep, including antigens based on the circulating viral strain in Norway. Thus, the assay is tailored for usage in the Norwegian sheep population. Although this work shows promising results, including repeatability, analytical sensitivity, and specificity, the diagnostic characteristics must be evaluated before the assay can be implemented in the Norwegian surveillance programme.

**Abstract:**

The Maedi-visna virus (MVV) causes a persistent infection in small ruminants, and its high genetic heterogeneity affects the performance of diagnostic tests when used in different populations. Therefore, the aim of this study was to develop a bead-based multiplex immunoassay tailored to detect antibodies against a Norwegian MVV strain. We used tissue samples from 14 PCR-positive sheep from a recent MVV outbreak in Norway to sequence the viral strain and produced recombinant antigens based on sequences from one animal. The assay included commercial TM-A and recombinant Norwegian p25, p16–25 and SU5 antigens. Cut-off values for each antigen were determined using receiver operating characteristic curves on 40 ELISA-negative and 67 ELISA-positive samples from the outbreak. The intraplate and interplate repeatability were investigated by testing a quadruplicate of five samples over three days, while the analytical sensitivity (aSe) and specificity (aSp) were measured in comparison to a commercial ELISA. The repeatability showed a coefficient of variation below 15% for most positive samples. The aSe was equal or higher for the multiplex assay than the ELISA, and the aSp of each antigen was 91.7, 93.3, 95.0 and 93.3% for p25, p16–25, SU5 and TM-A, respectively. The assay shows promising results; however, further evaluations of diagnostic characteristics are necessary before implementation in the Norwegian surveillance programme.

## 1. Introduction 

Maedi-visna is a chronic viral disease in small ruminants caused by Maedi-visna virus (MVV), a lentivirus in the family of *Retroviridae* [1]. The virus causes a persistent infection that may lead to the development of clinical signs from the respiratory tract or the nervous system, referred to as *maedi* and *visna*, respectively [2]. Maedi-visna is a notifiable disease in Norway, and a national surveillance program has been in effect since 2003. The disease had not been detected for fourteen years; however, between 2019 and 2020, MVV was diagnosed in nine flocks [3]. Preliminary analysis of selected viral segments showed the highest genetic similarities to the viral strain from an MVV outbreak in the same geographical area in 2002–2005.

There is no perfect test to diagnose an MVV infection [2,4,5]. Detecting the viral nucleic acid by PCR is challenging due to the low proviral load in infected animals in addition to the genetic heterogeneity of the virus. Therefore, serological tests are the most commonly used method to detect infected animals. However, serological tests also present challenges, including false negative results due to slow seroconversion, fluctuating antibody levels, high antigenic heterogeneity, and false positive results due to nonspecific binding or cross-reactive substances in serum [4,5]. The serological test used to detect and control MVV varies between countries, and a combination of tests is often used [6,7,8]. In the Norwegian surveillance programme, two commercial indirect enzyme-linked immunosorbent assay (ELISA) tests are used in a serial interpretation [3]. The Norwegian Food Safety Authority (NFSA) imposes restrictions on flocks with an MVV diagnosis. Thus, giving a flock an MVV diagnosis based on false positive test results can have severe consequences.

MVV is closely related to caprine arthritis and encephalitis virus (CAEV), and they are referred to together as small ruminant lentiviruses (SRLV) [9]. The SRLVs genome consists of the three structural genes *gag*, *pol* and *env*. The proteins encoded by *env* and *gag* have been shown to activate antibody-mediated adaptive immune responses against MVV [10,11]. During the early stages of infection, antibodies against gag proteins dominate the host’s humoral immune response. At later infection stages, the gag antibodies may decline, and antibodies against env proteins usually dominate [5]. Therefore, serological tests should include both gag and env antigens [12]. The *env* genes have greater variability than the *gag* genes, which are more conserved [4,10]. Due to the variability, especially in the *env* genes, the performance of a serological test in a sheep population will be influenced by how closely the viral strain antigens selected for the test match the proteins from the circulating viral strain. Immunodominant epitopes often used in serological tests are found in the capsid protein (CA, MVV-like p25 and CAEV-like p28) and the matrix protein (MA, p16), which are gag proteins, and in the transmembrane (TM) and surface protein (SU5), which are env proteins [13,14,15,16,17].

Bead-based immunoassays are capable of multiplex detection of antibodies targeted against various antigens with robust, sensitive data from a single well. The Luminex xMAP platform uses magnetic, fluorescently barcoded beads. Antigens are coupled to the beads, and fluorescence from phycoerythrin (PE) is used as a reporter for bound antibodies. One laser measures the fluorescence from the bead, while the reporter fluorescence is measured with a second laser. Each bead is barcoded with a unique concentration of two fluorescent dyes; thus, the inclusion of several different distinguishable beads enables the simultaneous evaluation of multiple analytes [18,19]. Therefore, for the detection of MVV antibodies, a bead-based multiplex immunoassay may discriminate between early and late infections if both env and gag antigens are included. Furthermore, antigens against other pathogens can be included in the same assay. Thus, multiplexing reduces the time, labour, and sample volume needed compared to running multiple ELISA tests. In addition, bead-based multiplex immunoassays have the potential to detect even lower amounts of analyte present in samples; thus, the analytical sensitivity can be higher than for ELISAs, as reported in various studies [20,21,22]. 

The World Organisation for Animal Health (WOAH) has developed an assay validation pathway divided into several stages [23]. Assessing the analytical characteristics is the first stage, and it includes evaluating the repeatability, analytical sensitivity (aSe), and specificity (aSp). The aSe is defined as the lowest amount of analyte present in a sample, resulting in a positive test result, while the aSp is defined as the ability of the assay to distinguish between the analyte and other non-target analytes. Assessing the diagnostic characteristics is the second stage of assay validation. The diagnostic sensitivity (dSe) is defined as the percentage of known infected animals that test positive in the assay, and the diagnostic specificity (dSp) is defined as the percentage of the known uninfected animals that test negative in the assay. The third and fourth stages include assessing the reproducibility and implementation of the assay. 

This study aimed to develop a bead-based multiplex immunoassay for the detection of antibodies against MVV and evaluate its analytical characteristics. The new assay includes proteins from the Norwegian viral strain, optimising it for use in the Norwegian sheep population.

## 2. Materials and Methods

### 2.1. Reagents

Inorganic chemicals and organic solvents used were reagent grade or better. Production of recombinant Norwegian antigens included the use of MagNA Pure External Lysis Buffer from Roche, Basel, Switzerland, a PCR clean-up extraction kit from Macherey-Nagel, Düren, Germany, restriction enzymes from Thermo Fisher Scientific, Waltham, MA, USA, pGEX-6P vector and glutathione sepharose 4B resin from Cytiva, Marlborough, MA, USA, and Quick Start Bradford protein assay from Bio-Rad, Hercules, CA, USA. After buffer exchange for some of the antigens (the Norwegian recombinant proteins and the commercial p25 and p16–25), protein quantification was performed using Qubit™ Protein Assay Kit from Thermo Fisher Scientific. For coupling of antigens, magnetic beads (MagPlex) and Bio-Plex Amine Coupling Kit from Bio-Rad, were used. Coupling of antigens with GST was confirmed using the anti-GST antibody clone vpg66 and streptavidin R-phycoerythrin (SAPE), both from Thermo Fisher Scientific. For running the immunoassay, protein G conjugated to phycoerythrin (protein G/PE) from AcZon Pharma, Bologna, Italy was used. During optimisation and verification, a sheep serum from In3diagnostic, Turin, Italy and a reference serum from Innovative Diagnostics, Grabels, France, were included.

### 2.2. Commercial Antigens

The commercial antigens p25, p16–25, and TM-A were received from In3diagnostic (Turin, Italy). P25 was a recombinant protein cleaved from GST moiety, p16–25 was a recombinant protein not cleaved from GST moiety, and TM-A was a synthetic peptide conjugated with bovine beta-casein. The p25 and p16–25 were based on the viral strain K1514 It561, while the TM-A sequence was based on a consensus of genotype A strains. The commercial p16–25 was a full-length antigen, while commercial p25 and TM-A were shorter immune-dominant epitopes within p25 and TM, respectively. The sequences of each of the commercial antigens are shown in Table S1 in the Supplementary Material.

### 2.3. Production of Recombinant Proteins Based on the Norwegian Viral Strain

The proteins p25, p16–25 and SU5 were based on a sequence from the Norwegian viral strain (Table S1), and the p25 and p16–25 proteins were designed on the full-length sequence encoding the p25 and p16–25 proteins, while the SU5 protein was an immune-dominant epitope within SU5. Frozen lung and lymph node tissue samples from 14 previously PCR-positive sheep from an outbreak in 2019 were used for DNA extraction. Briefly, <20 mg tissue sample was homogenised in MagNA Pure External Lysis Buffer using a tungsten carbide bead (Qiagen, Hilden, Germany) and the Mixer Mill (Retch, Haan, Germany). MagNA Pure 24 System (Roche, Basel, Switzerland) was used to isolate the DNA. The samples were PCR amplified using T100 Thermal Cycler (Bio-Rad). To retrieve 1.3 kb and 0.8 kb *gag* gene fragments, a nested PCR was performed on all samples using two forward primers and two reverse primers, as described in Grego et al. [24]. For SU5, all samples positive for 0.8 kb gag gene fragments were run in a hemi-nested PCR with the forward primers #563 and #567 and reverse primer #564, as described in Mordasini et al. [15]. PCR products were analysed using 1.5% agarose gel electrophorese and purified with a PCR clean-up gel extraction kit. All positive 0.8 kb *gag* gene fragments and SU5 fragments were sequenced using Sanger sequencing (BMR Genomics, Padova, Italy). To retrieve the whole *gag* gene sequences, two samples from one animal were subjected to amplicon sequencing using Nextera XT protocol, v2 chemistry (Illumina, San Diego, CA, USA) and Illumina Miseq platform. Based on the sequencing results, we designed two forward and one reverse primer to amplify the gene fragments encoding p25 and p16–p25 antigens. To insert the sequences into the pGEX-6P vector, the restriction enzymes BamHI and SalI were used to cut the vector, while BglII (giving BamH1-compatible cohesive ends) plus SalI were used to cut the inserts. The SU5 insert, including the BamH1 and EcoR1 restriction sites, was synthetically produced (BMR Genomics, Padova, Italy) and ligated into pGEX-6P plasmid vector. After purification, the vector and the inserts were purified and joined using standard T4 ligation. The expression of each recombinant protein followed the same protocol. The pGEX-6P vectors with our inserts were transformed into *Escherichia coli* (*E. coli*) strain BL21 C43 by 10 min incubation on ice followed by heat shock at 42 °C for 2 min and incubation with Luria-Bertani (LB) broth for 40 min with agitation at 37 °C. Thereafter, the transformed *E. coli* were grown on LB agar plates containing ampicillin (50 µg/mL) overnight. Selected colonies were screened by PCR to confirm that the *E. coli* bacteria contained plasmids with the desired inserts. The selected bacteria were grown overnight in LB broth containing ampicillin (100 µg/mL). Proteins were expressed by induction with Isopropyl β-D-1-thiogalactopyranoside followed by incubation for 2 h at 220 rpm at 37 °C. The bacteria were lysed using the conventional physico-chemical method, and the product of interest was purified from the soluble fraction by affinity chromatography using Glutathione Sepharose 4B resin, with elution buffer containing 10mM reduced glutathione at pH8. Quantification of purified proteins was performed using a Quick Start Bradford ProteinAassay. Sodium dodecyl-sulfate polyacrylamide gel electrophoresis (SDS-PAGE) analysis followed by staining with Coomassie blue was used to confirm that a protein of the expected size was located in the soluble fractions.

### 2.4. Serum Samples

Serum samples for this study originated from the Norwegian national surveillance programme for MVV during 2019–2023 and from the outbreak investigation performed by the Norwegian Food Safety Authority (NFSA) during the MVV outbreak in Norway in 2019–2020 [3]. During the outbreak investigation, all sheep flocks in the defined area were sampled and tested with a screening ELISA (ID Screen^®^ MVV/CAEV Indirect Kit (IDvet Grabels, France)) and positive samples were further tested with a verification ELISA (MVV/CAEV p28 Ab Verification Test (IDEXX Laboratories, Westbrook, ME, USA)), hereafter referred to as the screening and verification ELISA. Both ELISAs were performed according to the manufacturer instructions. Nine flocks were defined as MVV positive, three of them with a seroprevalence below 2% and with no positive PCR results, and the other six flocks with a seroprevalence above 30% and positive PCR results from one or more animals [25]. The samples were stored at −20 °C at the Norwegian Veterinary Institute (NVI). Serum samples were grouped into nine panels to optimise and validate the immunoassay (Table 1). Samples positive in the screening and verification ELISAs are hereinafter referred to as ELISA-positive, while samples negative in the screening ELISA are hereinafter referred to as ELISA-negative. Panel 9 (Table 1) included samples also analysed with Elitest MVV/CAEV (Hyphen Biomed, Neuville-sur-Oise, France), hereinafter referred to as Elitest. The Elitest was performed as recommended by the manufacturer.

### 2.5. Coupling of Antigen to Beads

The native buffers of the Norwegian recombinant proteins and the commercial p25 and p16–25 proteins were exchanged to phosphate buffered saline (PBS) using Vivaspin^®^ 500 (Sartorius, Göttingen, Germany) with molecular weight cut-offs of 30,000 and 10,000 [26]. The protein concentration after buffer exchange was quantified using the Qubit™ Protein Assay Kit (Thermo Fisher Scientific) according to the instructions stated by the manufacturer. The viral commercial antigens p25 and p16–25 were coupled to microspheres (MagPlex^®^-C, Bio-Rad) #29 and #54. The viral Norwegian recombinant antigens p25, SU5 and p16–25, and the commercial TM-A were coupled to microspheres #26, #37, #45 and #34, respectively; #29 was used as a BSA-coated control bead (blocked without being coupled to a protein), and GST alone was coupled to beads #54/55/64 (background control). The coupling was performed using the Bio-Plex Amine Coupling Kit according to the instruction manual. Briefly, the beads were re-suspended by vortexing for 30 s and sonication for 20 s. Then 1.25 × 10^6^ beads were washed with wash buffer using a magnetic separator before being activated with activation buffer, 10 µL of 50 mg/mL of Sulfo-N-hydroxysulfosuccinimide, and 1-ethyl-3-(3-dimethylaminopropyl)carbodiimide hydrochloride, followed by a 20 min incubation on a rotator (VWR^®^ Tube Rotator, Radnor, PA, USA) or shaker at 800 rpm (Eppendorf™ MixMate, Hamburg, Germany) at room temperature. After several washing steps with PBS, either 5 µg, 8 µg, or 12 µg of antigen were added, and the volume was brought to 500 µL with PBS before incubation for 2 h at room temperature on a rotator or on a shaker at 800 rpm. Then, following another washing step with PBS, the beads were incubated with a blocking buffer at room temperature for 30 min on a rotator or shaker. They were finally stored in the dark at 2–8 °C in 150 µL of storage buffer. The bead concentration was determined using TC20 Automated Cell Counter (Bio-Rad).

### 2.6. Bead-Based Immunoassay

The coupled beads were prepared by vortexing before diluting the beads in PBS+ buffer (PBS 1× with 0.5% bovine serum albumin and 0.5% sodium azide) to a final concentration of 50 beads/µL for each bead region. The assay was performed by adding 50 µL of the beads (~2500, singleplex, only one bead region) or bead mix (multiplex, multiple bead regions using 2500 beads per region) in solution to each well. The plate with the beads was washed three times with PBS containing 0.1% Tween (wash buffer) in Bio-Plex Pro Wash Station (Bio-Rad). Serum samples were diluted in PBS+, and 50 µL was added to each well. The plates were incubated on a shaker at 800 rpm in the dark for 60 min, then washed three times with wash buffer. Next, 50 µL of protein G/PE (1.25 mg/mL, diluted 1:500 in PBS+) was added to each well before incubation on a shaker at 800 rpm in the dark for 30 min. Finally, the plates were read in Bio-Plex 200 (Bio-Rad) and analysed using Bio-Plex Manager Software version 6.2. The signal was measured as median fluorescence intensity (MFI) of at least 100 beads per bead region, and the doublet discriminator gate was set to 5000–25,000. Each plate included two wells where serum samples were replaced with PBS+, designated as blank, and the blank signal was subtracted from the sample signal. Additionally, when samples were analysed in the multiplex format, the MFI signal from the BSA-coated control bead was also subtracted from the MFI signal from the beads coupled with antigens (except when the multiplex format was compared to the singleplex format, Figure S2) to exclude nonspecific reactions in the sample to the beads themselves (corrected MFI). Furthermore, each plate included two wells with the positive pooled sample and two wells with the negative pooled sample from panel 1 (Table 1) as positive and negative controls, respectively. The controls were used to calculate the sample-to-positive ratio (S/P%) using the following formulae:

$$S/P\%=\frac{MFIsample-MFInegativecontrol}{MFIpositivecontrol-MFInegativecontrol}*100$$

### 2.7. Coupling Confirmation

The coupling of the Norwegian recombinant proteins or GST to beads was confirmed using a biotinylated monoclonal anti-GST antibody, clone vpg66 and SAPE. The procedure was identical to the bead-based assay (described in Section 2.6), with the following exceptions: the first incubation lasted for 30 min at 750 rpm, SAPE was used instead of protein G/PE and diluted 1:50, and lastly, after the final incubation at 750 rpm, the wells were washed three times with wash buffer (PBS containing 0.1% Tween) and incubated with 100 µL sheath fluid on a shaker for 5 min prior to reading. The coupling of commercial antigens to beads was confirmed by the pooled samples in panel 1 (Table 1) and serum from a sheep immunised with a recombinant chimeric antigen derived from a fusion protein p25/TM-A (received from In3diagnostic).

### 2.8. Assay Optimisation

To assess the optimal coupling concentrations, each antigen was coupled to beads using three different amounts (5 µg, 8 µg, and 12 µg). The optimal coupling concentrations were selected as the amount of antigen showing the highest MFI signal-to-noise ratio (S/N) when serial dilutions of samples from panel 1 (Table 1) were run in singleplex.

Samples from panel 3 (Table 1) were 2-fold diluted (1:25 to 1:800) and analysed in multiplex. The optimal serum dilution showed a high S/N and, at the same time, exhibited a strong MFI response to all antigens. When the optimal serum dilution had been determined, the positive pooled sample from panel 1 (Table 1), together with one positive and one negative sample from panel 3 (Table 1), were also analysed in singleplex and compared to the multiplex results, to investigate possible antibody cross-reactivity to the specific antigens coupled to different beads.

The recombinant antigens were GST tagged; thus, to investigate whether animals had antibodies to GST that could potentially influence the results, beads coated with GST were only analysed with all serum samples from all nine panels.

### 2.9. Validation of Analytical Characteristics

To assess the analytical characteristics of the assay, a preliminary cut-off value for each antigen was determined by ROC curve analysis using GraphPad Prism version 9.3.1, with samples from panel 5 (Table 1). The samples were defined as positive (67) or negative (40) based on the ELISA results and were from flocks diagnosed with MVV and flocks without MVV diagnosis, respectively. Flocks were diagnosed with MVV by NFSA based on ELISA results (contact flocks) or a combination of ELISA and PCR results (non-contact flocks) [27]. The cut-off values were set to obtain maximum Se + Sp, in other words, where the curve is closest to the top-left corner of the graph. The area under the curve (AUC) was estimated from the ROC curve analysis for each antigen. 

To measure repeatability, the five samples in panel 4 (Table 1) were run in quadruplicate on each plate on three different days. Repeatability was determined as the percentage coefficient of variation (CV) of MFI values both within the assay (intraplate) and between assays (interplate).

The analytical sensitivity (aSe) was evaluated by investigating the limit of detection (LOD) compared to the LOD in the screening ELISA. Samples from panel 6 (Table 1) and an internal reference standard developed by Innovative Diagnostics [28] were 2-fold diluted from 1:25 to 1:102,400, assuring that the final sample volumes added to the multiplex assay and the screening ELISA were comparable. The highest dilution, giving a positive result for each antigen in the multiplex assay and for the screening ELISA was determined. 

Evaluation of analytical specificity (aSp) was performed using GraphPad Prism version 9.3.1 with the screening ELISA as a reference test and included 60 ELISA-negative samples from MVV negative flocks distributed throughout Norway (panel 7, Table 1). Five of these samples were haemolytic, and five were positive for *Toxoplasma gondii* when tested using an in-house antibody ELISA. 

### 2.10. Additional Testing of Samples

Samples from panel 8 (Table 1) were analysed with the bead-based multiplex immunoassay in order to investigate whether antibodies against the recombinant Norwegian antigens were detected from all flocks defined as positive with a prevalence above 30% during the outbreak in 2019–2020. 

In addition, samples with discrepant results in the three ELISA tests (panel 9, Table 1) were investigated with the multiplex assay to evaluate if the multiplex assay could determine if such samples are likely to be true or false positive. 

## 3. Results

### 3.1. Production of Recombinant Proteins

**Figure 1:**
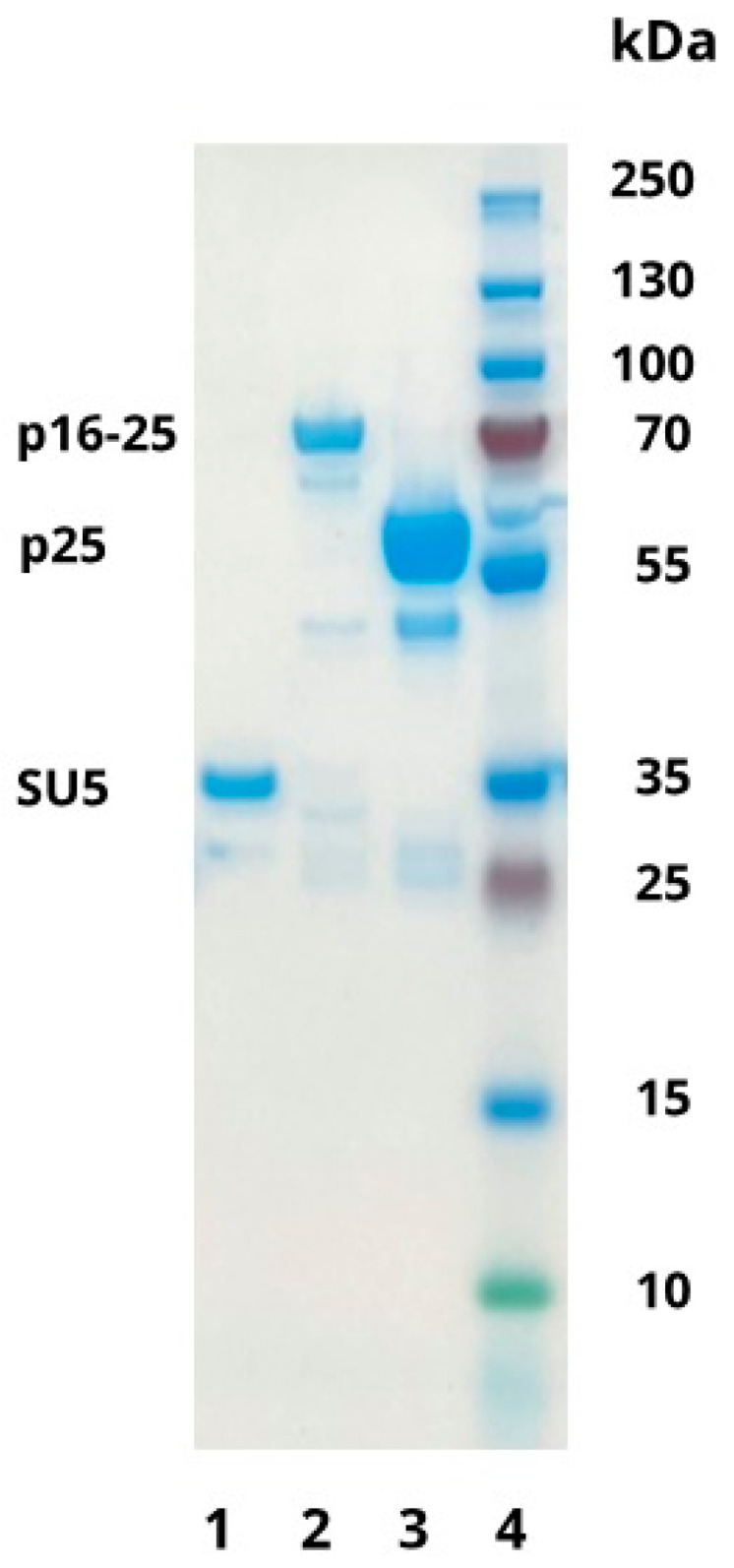
Coomassie blue staining of SDS-PAGE gel showing the Norwegian recombinant proteins with the expected molecular sizes of 29 kDa, 66 kDa, and 50 kDa for SU5 (lane 1), p16–25 (lane 2) and p25 (lane 3), respectively. The ladder is represented in lane 4.

The SDS-PAGE gel staining with Coomassie blue confirmed that the recombinant proteins based on the sequence of the circulating Norwegian viral strain had the expected molecular weights of approximately 66 kDA, 50 kDA, and 29 kDa for p16–25, p25, and SU5, respectively (Figure 1).

### 3.2. Development and Optimisation of the Bead-Based Multiplex Immunoassay

The successful coupling of the Norwegian recombinant proteins to beads was confirmed by a monoclonal anti-GST antibody, as high MFI signals were observed for all antigens tested compared to the negative control (Figure S1). The successful coupling of the commercial antigens was confirmed using the positive pooled serum sample (panel 1, Table 1) or a sample from a sheep immunised with a recombinant chimeric antigen derived from a fusion protein p25/TM-A, as the MFI of either of these two samples was higher than the MFI from the negative pooled sample (panel 1, Table 1) for all antigens (Figure S1).

**Figure 2:**
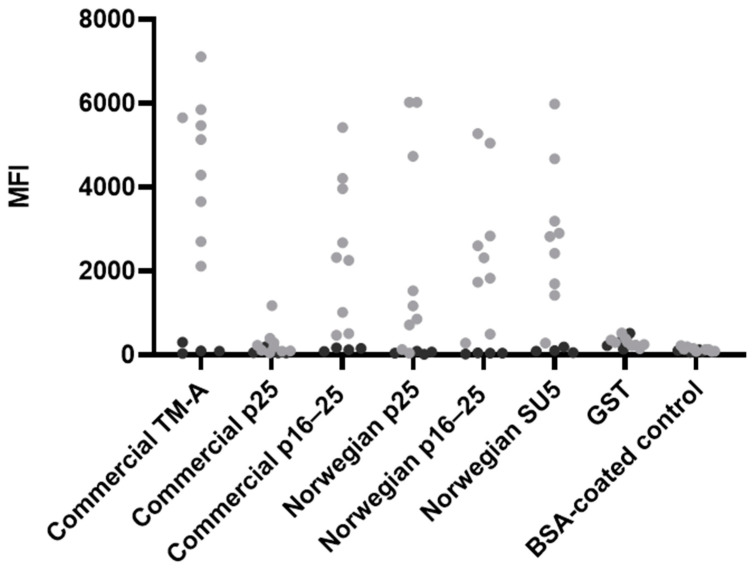
The median fluorescence intensity (MFI) of ELISA-positive samples (grey) and ELISA-negative samples (black) for all antigens and controls (GST and the BSA-coated control beads) in the bead-based immunoassay when MFI signal from the blank wells were subtracted. The samples were from panel two and were diluted 1:100. The amount of antigen coupled to beads was 8 µg for the TM-A antigen, the commercial p16–25, the Norwegian p16–25, and the Norwegian SU5 antigen, while 12 µg was coupled to the commercial p25, the Norwegian p25 and the GST-coated bead.

Initially, beads coupled to commercial antigens (TM-A, p25 and p16–25) were tested with serum samples from panel 2 (Table 1) in a 1:100 dilution. Testing of the commercial TM-A revealed a clear difference in the MFI levels of samples identified as positive and negative in ELISA. Compared to TM-A, the detection efficiency was lower for the commercially obtained p25 antigen and relatively similar for the p16–25 antigens (Figure 2). Therefore, recombinant p25, p16–25, and also SU5 proteins based on the Norwegian viral strain were produced. The detection efficiency of the Norwegian recombinant p25 and p16–25 appeared similar or better than the commercial antigens, and testing of the recombinant SU5 also generally revealed a difference in MFI levels between ELISA-positive and negative samples from panel 2 (Table 1, Figure 2). In order to include antigens optimised for use in the Norwegian sheep population, the commercial p25 and p16–25 antigens were excluded from further optimisation and evaluation of the assay. No difference between ELISA-positive and negative samples was observed for beads coated with GST and the BSA-coated control beads when analysed with samples from panel 2 (Figure 2). 

**Figure 3:**
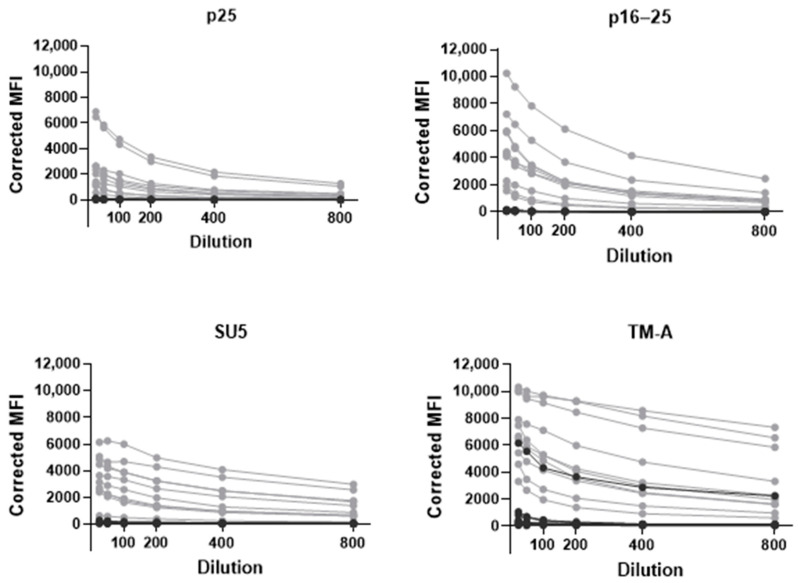
Corrected median fluorescence intensity (corrected MFI) in the bead-based immunoassay for the beads with the Norwegian recombinant antigens p25, p16–25 and SU5, and the commercial TM-A antigen, when tested with twofold dilutions (1:25 to 1:800) of samples from panel 3. Corrected MFI was the MFI signal after subtraction of the MFI signal from the blank wells and the MFI signal of the BSA-coated control bead. The grey dots represent ELISA-positive samples (10 samples), and the black dots represent ELISA-negative samples (8 samples).

The optimal amount of antigen coupled to beads was found to be the amount showing the highest S/N ratio when run in a singleplex with positive and negative pooled sera (panel 1, Table 1). The optimal amounts were 12 µg for the Norwegian recombinant p25, 8 µg for the Norwegian recombinant p16–25, 8 µg for the Norwegian recombinant SU5, 8 µg for the commercial TM-A and 12 µg for GST. After performing a serial dilution as described in Section 2.8 (Figure 3), the optimal serum dilution was found to be 1:100. 

The MFI signals from antigens coupled to beads and analysed either in singleplex or multiplexed format when samples were diluted 1:100 provided similar results, indicating no antibody cross-reactivity to the specific antigens coupled to different bead regions (Figure S2). For optimal comparison of singleplex and multiplex formats, the MFI signal from the BSA-coated control bead was not subtracted in the multiplex format. 

Approximately 6% of all samples (from all nine panels, Table 1) showed elevated MFI signal (above 1000 MFI) to the beads coated with GST only. Among the ELISA-negative samples, four of the samples with an MFI signal above 1000 for the GST-only bead were positive against one or two of the Norwegian recombinant proteins in the bead-based multiplex immunoassay. For the remaining ELISA-negative samples with an MFI signal above 1000 for the GST-only bead, the signal from the antigen-coupled beads was low compared to the signal from the GST-only bead (and were not classified as positive). 

### 3.3. Analytical Characteristics

**Figure 4:**
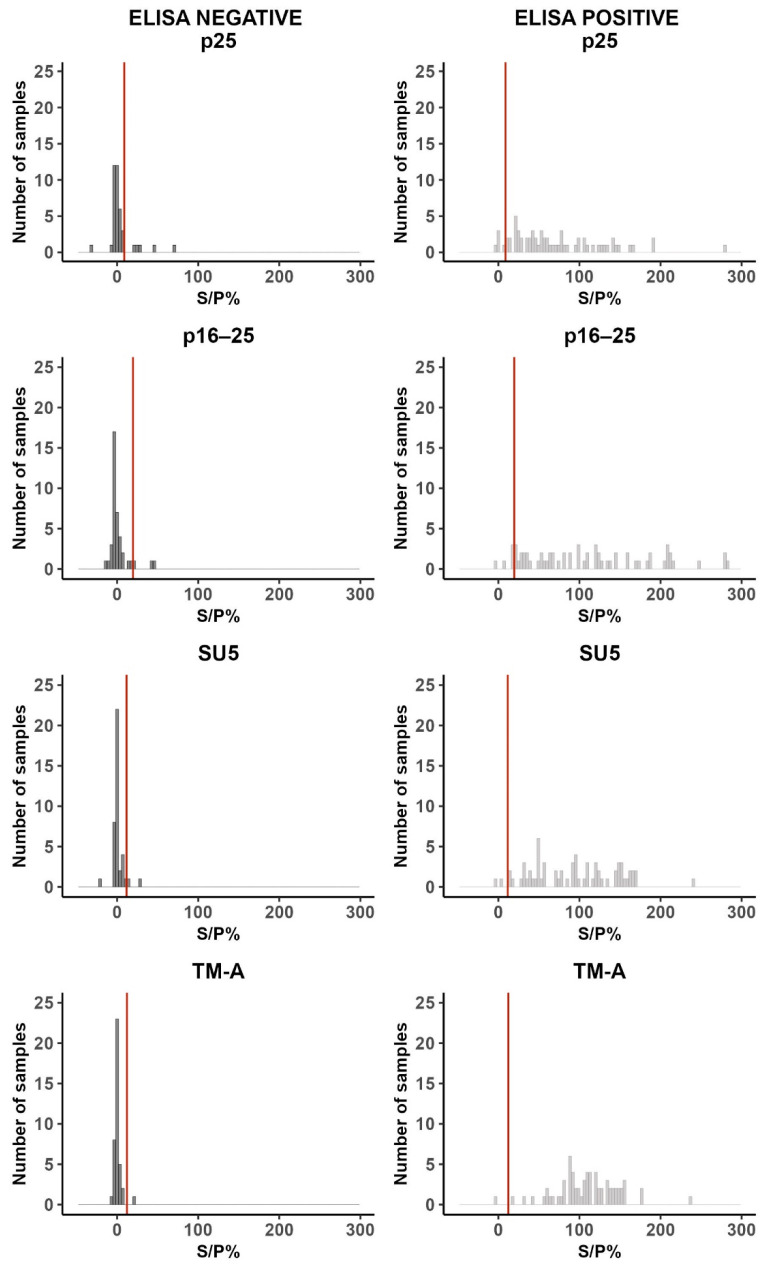
The distribution of S/P% for the Norwegian recombinant p25, p16–25 and SU5, and the commercial and TM-A antigen in the bead-based multiplex immunoassay when testing samples from panel 5. The black bars represent ELISA-negative samples (**left panel**), while the grey bars represent ELISA-positive samples (**right panel**). The S/P% values are shown on the *x*-axis, and the *y*-axis illustrates the number of samples within each S/P% value category (bin width 100). The cut-off values for each antigen are illustrated as red vertical lines.

The S/P% cut-off values were defined by ROC curve analysis as described in Section 2.9 and were 8.83, 19.60, 11.61, and 12.18 for the Norwegian recombinant antigens p25, p16–25 and SU5, and commercial TM-A, respectively (Table S2). The AUC were 0.93, 0.97, 0.98 and 0.99 for p25, p16–25, SU5 and TM-A, respectively (Figure S3 and Table S2). The estimated diagnostic sensitivity and specificity are shown in Table S2. Figure 4 shows the cut-off values and the sample distributions of samples in panel 5 (Table 1). Among the ELISA-positive samples from panel 5, five were below the cut-off value for p25, five were below the cut-off value for p16–25, two were below the cut-off value for SU5, and one was below the cut-off value for TM-A. Out of these, one sample was negative against both p25 and p16–25; one sample was negative towards all four antigens, while the rest were negative towards only one of the antigens. Among the ELISA-negative samples, five were above the cut-off value for p25, three for p16–25, two for SU5, and one for TM-A. Among these, one sample was above the cut-off value for all three Norwegian recombinant antigens; two were above the cut-off value for p16–25 and p25, while the rest were above the cut-off value for one antigen only.

Samples from panel 4 (Table 1) were investigated for repeatability. Each of the three intraplate repeatability runs towards the Norwegian recombinant p25, p16–25 and SU5, and the commercial TM-A antigen showed a CV below 15% for ELISA-positive samples except for one sample that was positive towards p25 (CV of 15.5%) (Table S3). However, the mean CV of intraplate repeatability was below 15% for all positive samples. Among ELISA-negative samples, five samples had a CV above 15% when evaluating each run for intraplate repeatability. When evaluating the mean CV of the three intraplate repeatability runs, only one negative sample still had a CV above 15% (Table S3). All ELISA-positive and negative samples had a CV below 15% towards the bead with GST only, both when evaluating each intraplate CV and the mean intraplate repeatability. The CV for intraplate repeatability for the BSA-coated control bead was above 15% for four and three samples when investigating each intraplate CV and mean intraplate, respectively. The interplate repeatability showed a CV below 15% for all positive samples except one (CV of 16.9% for beads coated with the p16–25 antigens), while the % CV showed a wider range for the inconclusive samples and negative samples (CV 2.7–38.6%) (Table S3). 

Table 2 shows the LOD observed for each of the antigens in the bead-based multiplex immunoassay and the screening ELISA. For the samples in panel 6 (Table 1), the bead-based multiplex immunoassay had an equal or lower LOD for all antigens compared to the screening ELISA. However, when the Innovative Diagnostics internal reference sample was tested, the bead-based multiplex immunoassay had a higher LOD towards p16–25 and SU5, compared to ELISA. 

**Figure 5:**
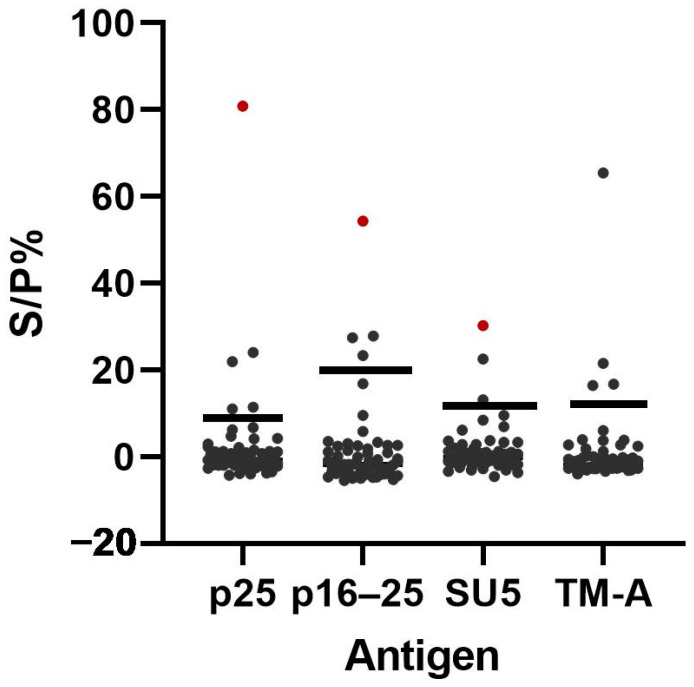
Distribution of S/P% for each antigen p25, p16–25, SU5 and TM-A in the bead-based multiplex immunoassay after analysis of 60 ELISA-negative samples (black dots). The red dots represent the same sample that was positive for all three antigens. The black horizontal lines represent the cut-off values for each antigen.

The aSp was determined by using the ELISA-negative samples from panel 7 (Table 1). Thirteen sera were positive for one or more antigens in the bead-based multiplex immunoassay. Two of them, either positive to p25 or TM-A only, were haemolytic samples. One sample, positive to p16–25 only, was a sample positive towards *Toxoplasma gondii* antibodies (in-house ELISA), while the other ten samples were samples from various geographical regions (north, east, south, or west of Norway). Table 3 shows the results for each antigen from the samples that were positive for one or more antigens. Figure 5 illustrates the distribution of S/P% values among the 60 ELISA-negative samples from panel 7. The aSp (95% confidence interval) for p25, p16–25, SU5, and TM-A was 91.7% (81.9–96.4), 93.3% (84.1–97.4), 95.0% (86.3–98.6), and 93.3% (84.1–97.4), respectively.

### 3.4. Additional Testing of Samples from High Prevalence Flocks

**Figure 6:**
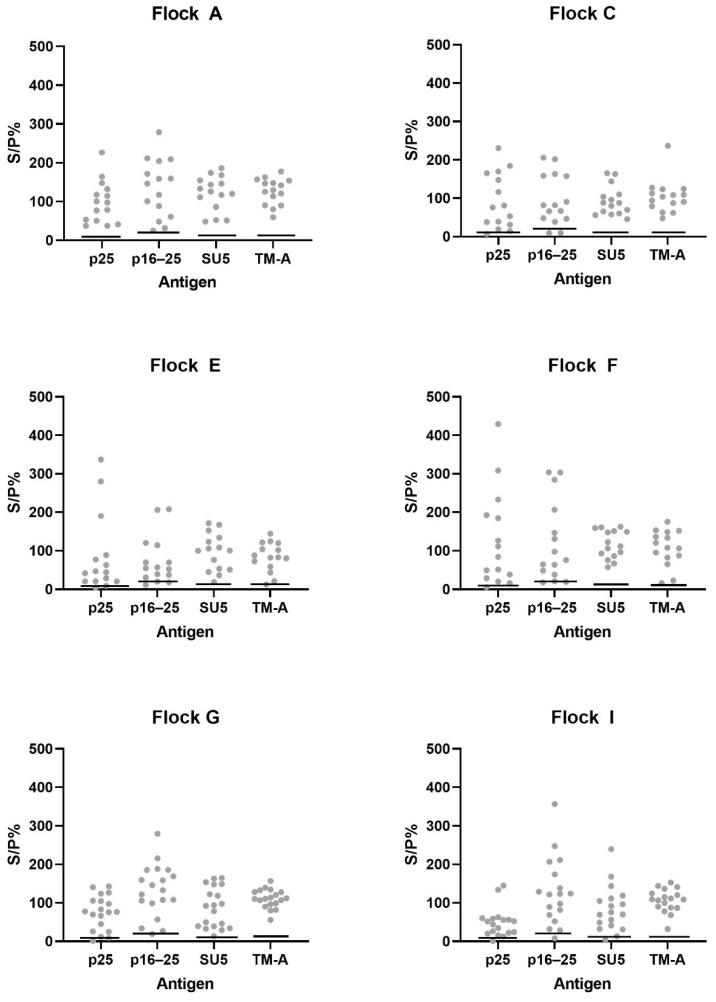
ELISA-positive samples from six flocks diagnosed with MVV in the 2019–2020 outbreak with a seroprevalence above 30% (15 samples from flocks A, C, E and F, 19 samples from flock G and 18 samples from flock I, from panels 5 and 8) were analysed by the bead-based multiplex immunoassay including antigens p25, p16–25, SU5 and TM-A. The flocks are defined as A–I, which corresponds to flock A–I in [25]. The horizontal lines represent the cut-off values for each antigen.

At least 15 ELISA-positive samples from each of the six flocks from the outbreak in 2019–2020 with a seroprevalence above 30% (from panel 5 and 8, Table 1) were analysed with the bead-based multiplex immunoassay, and the results are shown in Figure 6. For all of the flocks, the majority of the samples were above the cut-off value for all four antigens, and no obvious difference in antibody response (as seen by S/P%) between the six flocks was observed for either of the antigens.

### 3.5. Additional Testing of Samples with Discrepant ELISA Results

**Figure 7:**
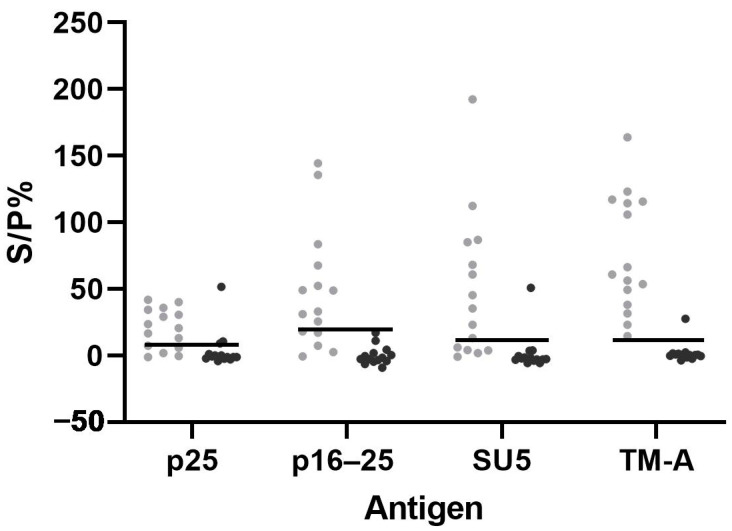
The S/P% from the bead-based multiplex immunoassay with antigens p25, p16–25, SU5 and TM-A, following the testing of samples positive in the screening and Elitest ELISA but negative in the verification ELISA (panel 9). The samples originated from flocks diagnosed with MVV (grey dots, 15 samples from 6 different positive flocks) and flocks without an MVV diagnosis (black dots, 14 samples from 12 different negative flocks). The horizontal lines represent the cut-off value for each antigen.

Figure 7 shows the S/P% from bead-based multiplex immunoassay testing of samples with discrepant results in the ELISA tests from positive and negative flocks (panel 9, Table 1). Among the fourteen samples from negative flocks, positive in screening and Elitest ELISA but negative in the verification ELISA, all but three samples were negative towards all antigens in the multiplex assay. Two of these were positive for p25 only, while one sample was positive for p25, SU5 and TM-A. Among the fifteen samples from positive flocks, eight samples were positive for all antigens, while none were negative for all antigens. Three samples were negative for all Norwegian recombinant proteins and only positive towards TM-A, while the other samples were positive for two or more antigens.

## 4. Discussion

Maedi-visna is a notifiable disease in Norway, and the aim is to eliminate the disease in the country. Currently, a serial interpretation of two commercial ELISA tests is used in the surveillance program and for diagnostic purposes. However, challenges with the interpretation of the results can arise when the two tests show discrepant results [25]. An assay tailored towards the circulating viral strain in Norway may improve serological detection, as commercial ELISA tests might not be equally applicable in different countries due to the SRLV’s high mutation rates [24]. Bead-based multiplex immunoassays allow simultaneous detection and differentiation between antibodies specific to several antigens, thus reducing the time, labour, sample material, and sample handling needed compared to running multiple ELISA tests [18,19]. The methodology can be used both to detect antibodies against multiple infectious agents in the same assay [29] and to detect antibody responses to several individual epitopes from the same agent [30,31]. As a bead-based multiplex immunoassay measures the reaction towards each antigen, the assay can potentially discriminate between early and late infections of MVV. This might be particularly useful information in flocks where MVV has not previously been detected. Furthermore, antigens towards other pathogens or SRLV strains, such as CAEV, might be added to the assay later on. In this study, we have therefore developed, optimised, and assessed the analytical characteristics of a bead-based multiplex immunoassay based on the Luminex platform detecting antibodies towards p25, p16–25, SU5, and TM-A antigens from MVV. To our knowledge, this is the first work describing the development of a bead-based multiplex immunoassay detecting MVV antibodies in sheep. Only one previous study describes the development of a bead-based multiplex immunoassay detecting antibodies towards SRLV in addition to two other infectious diseases [32]. That study included recombinant p16 and gp38 (TM) antigens and investigated 90 positive and 90 negative SRLV samples (defined by a commercial ELISA from Veterinary Medical Research and Development, VMRD, USA) with their multiplex assay. The authors reported a diagnostic Se and Sp of 84% and 95% for SRLV, respectively. However, this assay was developed and evaluated using goat samples from the goat sera bank in the Veterinary School of the National Autonomous University of Mexico; hence, this assay would not be optimal for use in the Norwegian sheep population.

Previous studies have investigated immunodominant epitopes of MVV and have identified epitopes of TM, SU5 and p25 as good candidates for usage in serological tests [13,14,15,16,17]. Antibodies towards gag and env antigens are produced at different stages of infection [11,33,34]. To diagnose MVV in sheep at different stages post-infection, serological assays should, therefore include both gag and env antigens. Initial testing of antigens for the bead-based multiplex immunoassay included commercial TM-A, p25 and p16–25 antigens and newly developed recombinant p25, p16–25, and SU5 antigens based on a viral strain isolated from an infected sheep during the last MVV outbreak in Norway in 2019–2020. During the assay development, antibody reactions towards each antigen were measured; thus, only antigens showing promising results were included in the further evaluation. Positive and negative samples from the Norwegian outbreak in 2019–2020 showed clear separation of MFI levels for the antibody response to the commercial TM-A antigen, while the separation was less visible for the commercial p16–25, and in particular for p25. The separation of MFI signals for the antibody response against the Norwegian recombinant p25 and p16–25 antigens appeared better than or similar to the commercial antigens (Figure 2). The commercial p25 antigen consists of an immune-dominant epitope; this sequence is identical in the Norwegian recombinant p25 antigen; however, the Norwegian recombinant p25 antigen consists of the full sequence of p25 as well. The commercial and Norwegian recombinant p16–25 are both full-length sequences. Based on our results, we excluded the commercial p25 and p16–25 from further development of the assay, while the Norwegian recombinant p25 and p16–25 were included for further development. SU5 is a well-known strong immunogen but highly strain-associated due to antigenic drift and is thus suited for serotyping [13]. Therefore, only a recombinant SU5 antigen based on the Norwegian viral strain was tested. The response towards SU5 revealed a separation of MFI levels between ELISA-positive and negative samples, as seen in Figure 3, and thus, we included it in the assay. By including antigens based on the Norwegian viral strain with promising results, the assay is optimised for use in the Norwegian sheep population. 

As described by WOAH, the first step in the validation pathway includes assessing the repeatability of the assay, as well as the analytical sensitivity and specificity [23]. Repeatability is a measure of the ability of an assay to produce similar results for multiple runs of the same sample [23]. WOAH recommends the use of CV to measure repeatability and that neither intra- nor interplate CV should exceed 15%. Exceptions from this recommendation are negative and low positive samples, which may have higher CVs [35]. The vast majority of our results comply with the recommendation from WOAH, and the repeatability of the bead-based multiplex immunoassay was regarded as acceptable (Table S3). Another recent bead-based multiplex immunoassay study reported CVs up to 20.5% for their positive control [32].

To evaluate the aSe and aSp, a preliminary cut-off value was defined for each antigen from the samples in panel 5 by defining samples as positive or negative based on ELISA results. The estimated diagnostic sensitivity and specificity, as well as the predictive values for the definition of ELISA-positive and negative samples when evaluated in the Norwegian sheep population, are described in Jerre et al. [36]. Although misclassification of samples might have occurred, no perfect reference test to detect MVV infection exists; thus, misclassifications would have been possible if other tests had been used as well [5]. The screening and verification of ELISA includes a mix of peptides from TM, SU and p25 antigens and TM and p28 antigens, respectively [28,36]. We regarded an ELISA-positive sample as positive towards all antigens in the multiplex assay. However, for the ELISA, we did not know if the samples were positive for only one or all antigens in the test. In the multiplex assay, on the other hand, the reaction to each antigen is measured. Hence, using the ELISAs to define samples as positive or negative might lead to wrong conclusions regarding the performance of the specific antigens in the multiplex assay. The overlap of MFI levels from positive and negative samples was greater for the gag antigens (p25 and p16–25) than the env antigens (SU5 and TM-A) (Figure 4), which can be due to a decrease in antibody levels towards the gag antigens in animals who have been infected for a long time. The 67 ELISA-positive samples (panel 5) used to define the optimal cut-off values originated from positive flocks from the 2019–2020 outbreak. Six out of the nine infected flocks had a seroprevalence above 30%, and it is therefore likely to assume that the flocks had been infected for a long time [25]. Both younger and older animals from these flocks were analysed, thus increasing the likelihood of including both recently infected animals and animals that had been infected for a while. Only one of the ELISA-positive samples was negative towards TM-A antibodies, and as these antibodies are produced at later stages of infection, this sample might come from a recently infected animal. In addition, the viral strain(s) used in the ELISAs are not publically distributed; hence, differences between the antigens used in the ELISAs and our multiplex assay are likely to have resulted in discrepancies between the two assays. 

The aSe was evaluated by comparing the LOD of the bead-based multiplex immunoassay with the screening ELISA. LOD is the lower limit of detection, which estimates the lowest amount of analyte that would produce a positive result [23]. The LOD was lower or equal for all antigens in the bead-based multiplex immunoassay when Norwegian samples were tested; thus, the aSe is improved in the multiplex as compared to the ELISA (Table 2). Interestingly, the Innovative Diagnostics internal reference sample showed a higher LOD for the bead-based multiplex immunoassay than the screening ELISA. Information regarding which viral subtype was used to produce the Innovative Diagnostics internal reference sera is not publicly distributed; thus, our finding might be due to a poorer match between the antibodies in the reference serum and the antigens used in the multiplex assay. 

The aSp of an antibody detection assay is the ability to distinguish the target analytes from other non-target analytes [23]. Cross-reactivity or nonspecific binding can result in false positive test results and should be investigated [35]. To evaluate aSp, 60 ELISA-negative samples were analysed with the multiplex assay (Table 3 and Figure 5). We wanted to include samples most likely to cause false positive reactions. However, the only known false positive reaction for SRLV antibody assays is seen in animals vaccinated against blue tongue virus due to immune responses against nonspecific proteins originating from antigen production in eukaryotic cell systems [37]. Vaccination against blue tongue virus is not performed in Norway; thus, the negative samples consisted of samples distributed in different geographical areas in Norway, in which samples with haemolysis and samples from animals with other known infections (positive in an in-house antibody ELISA against *Toxoplasma gondii*) were included, as both may cause false reactions. Eleven samples were positive against one of the four antigens, one sample was positive against two (p25 and p16–25), and one sample was positive for all three recombinant antigens from the circulating viral strain (Table 3 and Figure 5). As the aSe was higher in the multiplex than in the screening ELISA, we cannot exclude that some of these samples were true positives. In particular, the ELISA-negative sample that was positive for all three recombinant antigens could be a recently infected animal that had not yet produced TM-A antibodies [11]. The aSp of the multiplex assay when the screening ELISA was used as a reference test were 91.7% (81.9–96.4), 93.3% (84.1–97.4), 95.0% (86.3–98.6) and 93.3% (84.1–97.4) for p25, p16–25, SU5 and TM-A, respectively.

The full sequences of p25, p16–25 and SU5 could only be obtained in samples from one animal, and the recombinant proteins are based on these sequences. If there is more than one circulating viral strain in Norway, this might be a problem, especially as far as the SU5 antigen is concerned. Therefore, to assess whether this is of concern or not, a minimum of fifteen ELISA-positive samples from each of the six flocks from the outbreak with an MVV prevalence above 30% were analysed with the bead-based multiplex immunoassay. In each flock, the majority of samples were positive towards all antigens (Figure 6). Hence, there does not seem to be any major difference in the antibody reaction towards the recombinant antigens between flocks, suggesting a clonal origin of the outbreak. Hence, the recombinant antigens produced seem suitable for use in the assay, even though they all originate from a single individual. MVV has been under national serological surveillance since 2003, and MVV had not been detected in Norway for fourteen years until it was detected in 2019–2020 in the same area as last time [3]. Additionally, there are strict regulations on the importation and movement of live sheep in Norway [38]. Therefore, if a new outbreak occurs, it would most likely arise from the same circulating strain as seen in 2019–2020 instead of being a different strain. Thus, we believe that the developed assay will be suitable for future outbreaks of MVV in the country. However, should a new outbreak occur, the virus will be sequenced and compared to the sequences used in the multiplex assay.

As the verification ELISA is no longer available, the Elitest ELISA is used for verification in Norway. This has resulted in more positive samples in the serial interpretation of two ELISA tests, as well as flocks assumed to be negative based on historical data of MVV in Norway. Panel 9 included samples positive in the screening and Elitest ELISA but negative in the verification ELISA from MVV positive and negative flocks. Interestingly, most samples from positive flocks were above the cut-off values for all antigens in the bead-based multiplex immunoassay, while only two samples were above the cut-off for one antigen, and one sample was above the cut-off value for three antigens from the negative flocks (Figure 7). Although the true disease status of the tested animals is unknown, historical data on MVV in Norway has shown a low prevalence of MVV in the sheep population, and MVV was not detected in Norway between 2005 and 2019 [3]. Thus, samples positive in the screening and Elitest ELISA but negative in the verification ELISA, from assumed negative flocks, are most likely false positive in ELISA, while in positive flocks, such animals are more likely true positive. Therefore, the multiplex assay shows promising results in terms of being a useful assay to clarify assumed false positive ELISA samples. 

The recombinant antigens included GST, which can pose a problem as some sheep may have anti-GST antibodies [39]. Only approximately 6% of the animals tested in this study showed elevated MFI results towards the beads coated with GST only, and GST appeared to result in an unexpectedly elevated MFI signal from the recombinant proteins in four samples only. Preliminary testing of adding GST in excess to the serum dilution buffer in order to preadsorb potential anti-GST antibodies, as performed by Nogarol et al. [40], has shown promising results and will be included in the further development of the assay. Thus, adding an excess of GST in the serum dilution buffer could improve the aSp for the recombinant antigens. 

In this study, we have evaluated the analytical characteristics of the bead-based multiplex immunoassay under development. As described by WOAH, further steps in the validation pathway are to assess the diagnostic characteristics, including defining the optimal cut-off values and evaluating the diagnostic sensitivity and specificity [23]. There is no perfect test to diagnose MVV infection, and the true disease status of animals is unknown. Using another imperfect assay as a reference test, such as commercial ELISAs, for estimating the diagnostic sensitivity and specificity of the new multiplex assay can lead to biased results [41]. In the absence of a perfect reference test, WOAH recommends using latent class models to estimate the diagnostic characteristics [23]. Therefore, estimates of diagnostic sensitivity and specificity based on the ROC curves using the commercial ELISAs as reference tests (included in Table S2) might be inaccurate and should be re-evaluated using latent class analysis. Although the analytical characteristics of the assay developed in this study show promising results, further investigation of the diagnostic characteristics is necessary before the assay can be used for diagnostic purposes and implemented in the Norwegian surveillance program. The latent class analysis should also include evaluations of how a positive sample is defined and whether antibodies towards one, two or three antigens should be required. 

## 5. Conclusions

In this study, we have developed a bead-based multiplex immunoassay to detect antibodies towards the Maedi-visna virus. The assay included antigens from a viral strain circulating in Norwegian sheep in an outbreak in 2019–2020 and should, therefore, be suited for diagnostic purposes and surveillance in Norway. The assay included four antigens coupled to beads; the antigens were a commercial TM-A antigen as well as recombinant p25, p16–25 and SU5 antigens based on the Norwegian viral strain and one BSA-coated control bead. Analytical characteristics of the assay were investigated, showing repeatability in compliance with the recommendations from WOAH, an aSe equal to or higher than a commercial ELISA test, and an aSp ranging from 91.7–95.0% for each of the various antigens.

## Figures and Tables

**Table 1:** Panels of serum samples from Norwegian sheep were used for the optimisation and validation of a bead-based multiplex immunoassay for the detection of antibodies to Maedi-visna virus.

Panel	Samples	Usage
Panel 1	1 positive pooled sample (30 animals positive in screening and verification ELISA) 1 negative pooled sample (30 animals negative in screening ELISA)	Optimisation (antigen concentrations)
Panel 2	9 positives in screening and verification ELISA 4 negatives in screening ELISA	Optimisation (commercial and Norwegian antigens)
Panel 3	10 positives in screening and verification ELISA 8 negatives in screening ELISA	Optimisation (serial dilution)
Panel 4	5 samples with S/P% values ranging from positive to inconclusive and negative in screening ELISA	Repeatability
Panel 5	40 negatives in screening ELISA 67 positives in screening and verification ELISA	Cut-off determination
Panel 6	3 samples positive in screening and verification ELISA from panel 3	Analytical sensitivity
Panel 7	60 negatives in screening ELISA (50 evenly distributed throughout Norway, 5 haemolytic, and 5 positives in an in-house antibody ELISA for *Toxoplasma gondii*)	Analytical specificity
Panel 8	32 positives in screening and verification ELISA from positive flocks	Exploring samples from high prevalence flocks
Panel 9	15 positives in screening ELISA and Elitest, negative in verification ELISA from positive flocks 14 positives in screening ELISA and Elitest, negative in verification ELISA from negative flocks	Exploring samples with discrepant ELISA results

**Table 2:** The estimated limit of detection (LOD) following the serial dilution of three samples (panel 6) in the bead-based multiplex immunoassay and the screening ELISA. The LOD of an internal reference serum from Innovative Diagnostics (IDvet) was also examined. The sample-to-positive ratio (S/P%) at each specific dilution is shown in parenthesis. The S/P% cut-off value of a positive sample for the ELISA, as stated by the manufacturer, is 60 (the S/P% value of 50–60 is regarded as inconclusive and negative if the S/P% value is below 50).

Antigen/Test	Samples Panel 5 ^1^			IDvet Reference Serum (S/P%)
	Sample 1 (S/P%)	Sample 2 (S/P%)	Sample 3 (S/P%)	
ELISA	1:800 (112.9)	1:400 (87.3)	1:200 (68.4)	1:400 (62.2)
Multiplex p25	1:800 (14.4)	1:6400 (9.1)	1:1600 (12.6)	1:400 (13.9)
Multiplex p16–25	1:800 (35.5)	1:1600 (23.5)	1:400 (29.3)	1:50 (29.3)
Multiplex SU5	1:800 (16.5)	1:6400 (14.8)	1:6400 (13.6)	1:100 (12.1)
Multiplex TM-A	1:12,800 (14.0)	1:3200 (18.9)	1:800 (17.2)	1:1600 (18.4)

^1^ When analysed in the optimal sample dilution (1:100), the S/P% values for sample 1 were 58.8, 57.4, 140.3 and 81.6, for sample 2 the S/P% values were 214.9, 144.8, 157.6 and 105.2, and for sample 3 the S/P% values were 68.0, 76.5, 122.5 and 38.2 for the p25, p16–25, SU5, and TM-A antigens, respectively.

**Table 3:** The qualitative results (positive (+) or negative (−)) towards each of the antigens for the thirteen samples from panel 7 (60 ELISA-negative samples) that had a positive result in the bead-based multiplex immunoassay.

Sample Description	Antigen			
	P25	P16–25	SU5	TM-A
Haemolytic	+	−	−	−
Haemolytic	−	−	−	+
Toxoplasmosis +	−	+	−	−
East	−	−	−	+
East	+	−	−	−
East	−	−	+	−
East	+	+	−	−
South	+	−	−	−
South	−	−	−	+
West	+	+	+	−
West	−	+	−	−
North	−	−	+	−
North	−	−	−	+

## Data Availability

The original contributions presented in the study are included in the article/Supplementary Material; further inquiries can be directed to the corresponding author.

## Supplementary Materials

The following supporting information can be downloaded at: https://www.mdpi.com/article/10.3390/ani14101442/s1, Table S1: The sequence of each antigen used in the bead-based multiplex immunoassay under development; Figure S1: Coupling confirmation; Figure S2: Comparison of the median fluorescence intensity (MFI) of the singleplex and multiplex; Figure S3: The ROC curves for each antigen; Table S2: The area under the curve (AUC), cut-off values and diagnostic sensitivity (dSe) and specificity (dSp) estimated using the ROC-curves; Table S3: The coefficient of variation (CV) for all antigens in the bead-based multiplex immunoassay under development.
