# Supplementary material for: Development of a Bead-Based Multiplex Fluorescent Immunoassay to Detect Antibodies against Maedi-Visna Virus in Sheep
Source: Animals (Basel). 2024 May 12;14(10):1442. doi: 10.3390/ani14101442 (PMC11117221; doi:10.3390/ani14101442)
Supplement: Supplementary file 1 [file animals-14-01442-s001.zip › animals-2929894-supplementary.pdf]

Supplementary material

Table S1. The sequence of each antigen used in the bead-based multiplex immunoassay under development.

Antigen	Sequence
**Commercial**	
p16–25	MAKQGPKEKKGYPELRDVIKMTCKIKVGPGKETLTEGNCLWALKTTDFIFED<br>IKTEPWTITKMYTVWGRLKNLTPEETSKREFASLQATLACLMCSQMGMKPET<br>VQAARGIIEMKEGIQEKKTSENADKMERLYPNLEEYKQVYPIVNLQQGGRSWK<br>AVDSVVFQQSQTVAMQHGLVSEDFERQLAYYATTWTSKDILEVLAMMPGN<br>RAQKELIQGKLNDEAERWVRQNPPGPNVLTVDQIMGVGQTNQQAAQGNTD<br>QARQICLQWVINALRSVRHMSHKPGNPMLVKQKNSESYEDFIARLLEAIDAE<br>PVTDPIKTYLKVTLSYTNASTDCQKQMDRVLGQRVQQATVEENMQACRNV<br>GSE
p25	KELIQGKLNEEAERWVRQNPPGP
TM-A	ELDCWHYQHYCVTS
**Norwegian**	
p16–25	MAKQGSKEKKGYPELKEVIKATCKIKVGAGKETLTEGNCLWALKTIDFIFED<br>ITTEPWTLTKMYTVWSRLKQLTPEETSKREFASLQATLACIMCSQMGMKPET<br>VQAARGIISMKEGLQEIKEDKEKKVEQLYPNLERHREAYPIVNLQAGGRSWK<br>AVESVTFQQLQNVAMQHGLVSEDFERQLAYYATTWTSKDILEVLAMMPGN<br>RAQKELIQGKLNEEAERWVRQNPPGPNVLTVDQIMGVGQTNQQASQANMD<br>QARQICLQWVITALRSVRHMSHRPGNPMLVKQKNNESYEEFIARLLEAIDAE<br>PVTDPIKTYLKVTLSYTNASTDCQKQMDRVLGARVQQATVEEKMQACRDV<br>GSE
p25	PIVNLQAGGRSWKAVESVTFQQLQNVAMQHGLVSEDFERQLAYYATTWTS<br>KDILEVLAMMPGNRAQKELIQGKLNEEAERWVRQNPPGPNVLTVDQIMGV<br>GQTNQQASQANMDQARQICLQWVITALRSVRHMSHRPGNPMLVKQKNNES<br>YEEFIARLLEAIDAEPVTDPIKTYLKVTLSYTNASTDCQKQMDRVLGARVQQ<br>ATVEEKMQACRDVGSE
SU5	RVRAYTYGVIEMPESYSKSNRGKRS

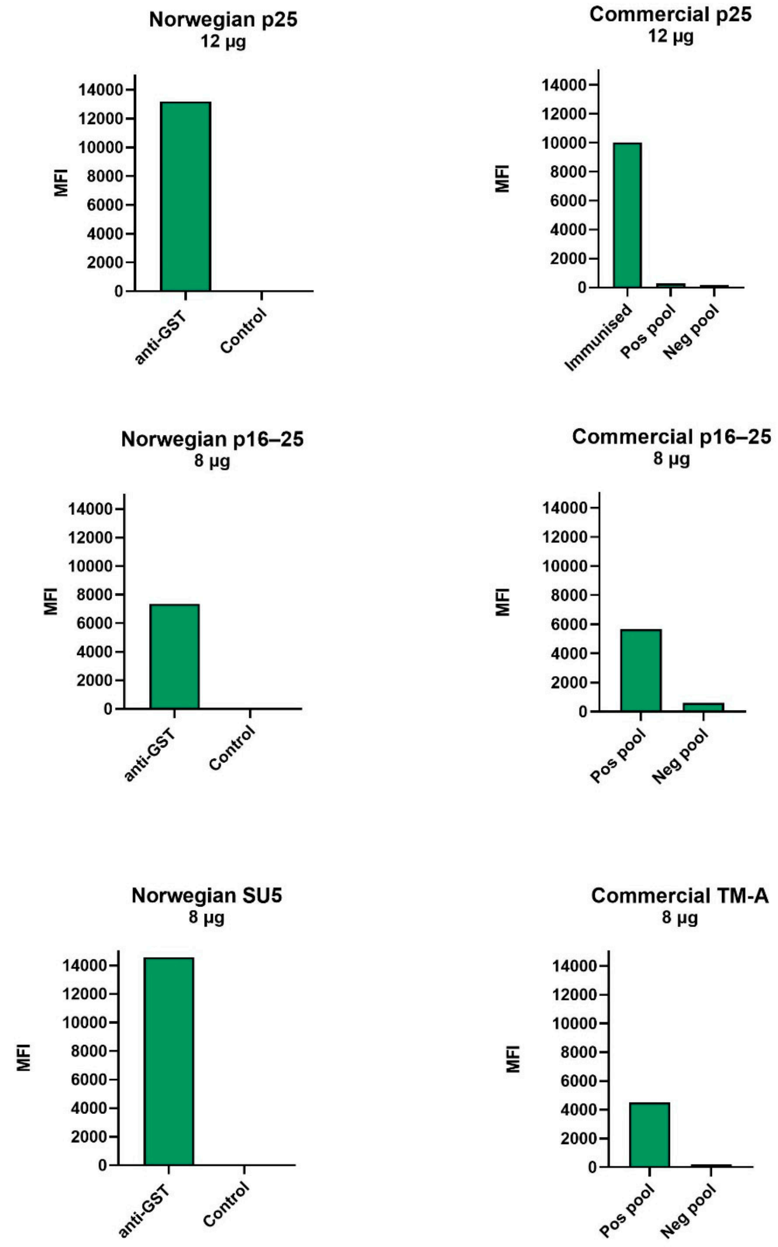

Figure S1. Coupling confirmation. The coupling of the recombinant Norwegian antigens (p25, p16–25 and SU5) were validated using monoclonal anti-GST antibodies (anti-GST) in 1:50 dilution compared to control (without adding the anti-GST). Whereas the coupling of the commercial antigens (p25, p16–25 and TM-A) were validated by comparing the positive pool (pos pool) and negative pool (neg pool) sample or the sample from an immunised sheep (immunised) diluted in 1:100. Only the optimal amount of antigen used in the final immunoassay are shown.

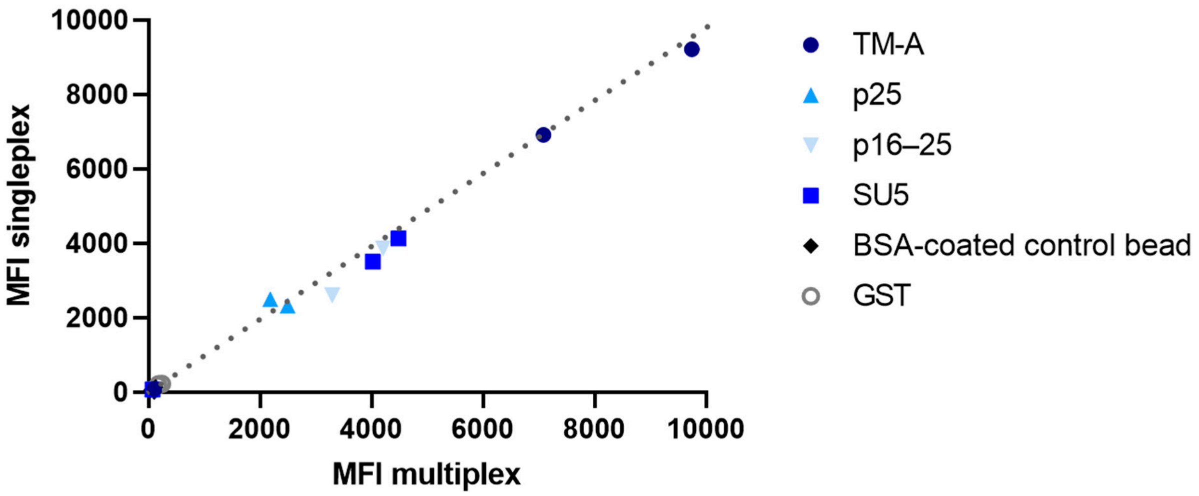

Figure S2. Comparison of median fluorescence intensity (MFI) of singleplex and multiplex. The MFI from singleplex (y-axis) and multiplex (x-axis) analysis after subtracting the MFI signal from the blank wells of the positive pooled sample from panel 1, and one positive and one negative sample from panel 2 diluted in 1:100. The dotted grey line shows how the distribution of MFI signal from singleplex and multiplex analysis would be if the results were identical.

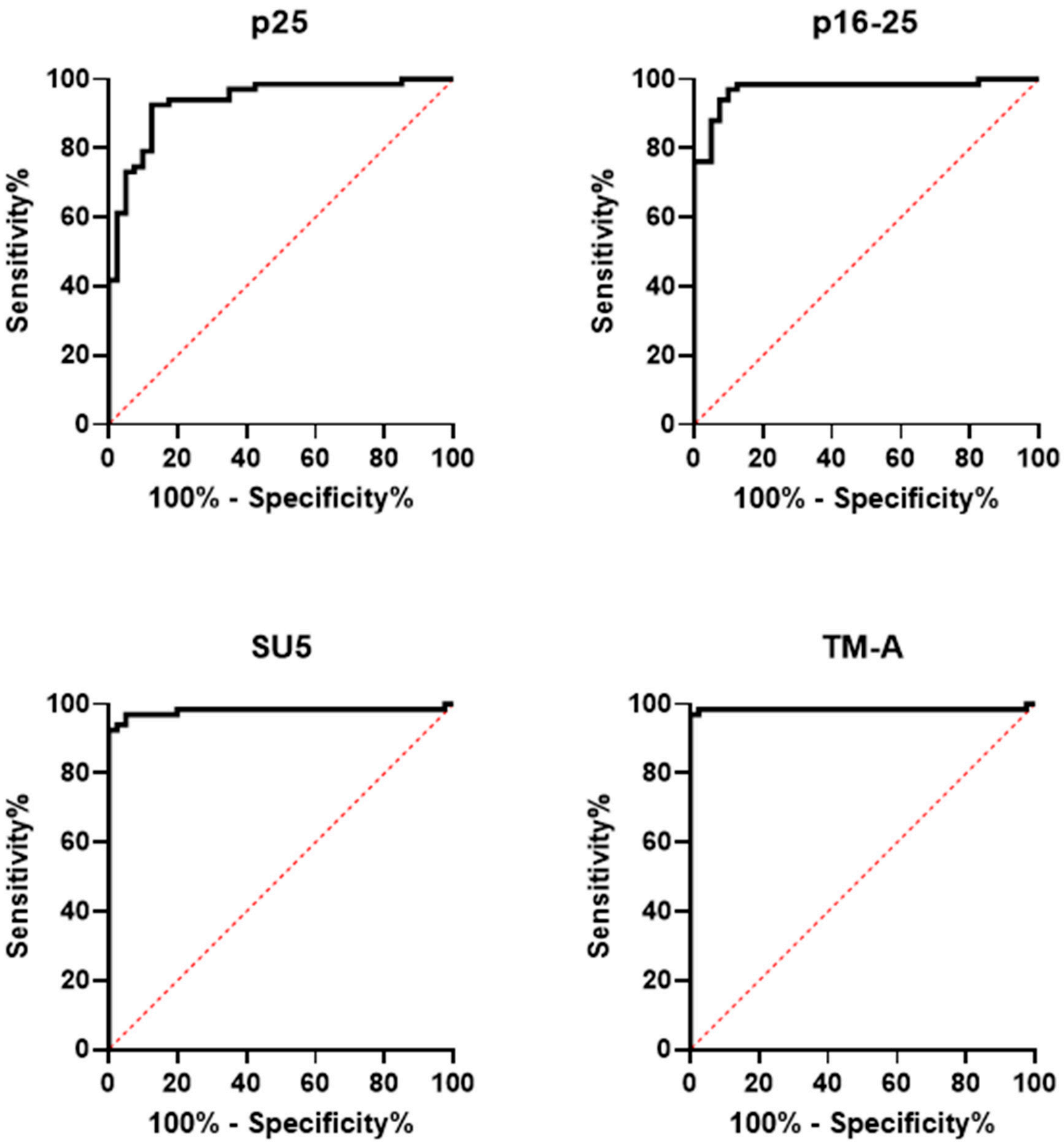

Figure S3. The ROC curves for each antigen. The cut-off values were defined as the value showing the maximum Se+Sp which is the at the top-left corner of each graph. The red dotted lines represents a test with a discriminating ability that is no better than chance alone.

Table S2. The area under the curve (AUC), cut-off values and diagnostic sensitivity (dSe) and specificity (dSp) estimated using the ROC-curves for the bead-based multiplex immunoassay.

Antigen	AUC	Cut-off	dSe	(95% CI)	dSp	(95% CI)
p25	0.93	8.83	92.5%	(83.7–96.8)	87.5%	(73.9–94.5)
p16–25	0.97	19.60	92.5%	(83.7–96.8)	92.5%	(80.1–97.4)
SU5	0.98	11.61	97.0%	(89.8–99.5)	95.0%	(83.5–99.1)
TM-A	0.99	12.18	98.5%	(92.0–99.9)	97.5%	(87.1–99.9)

Table S3. The coefficient of variation (% CV) for all antigens in the bead-based multiplex immunoassay under development. Five samples (panel 4) ranging from positive to inconclusive and negative in screening ELISA were used. The inconclusive sample was inconclusive in the screening ELISA and negative in the verification ELISA, and negative towards all antigens in the multiplex assay. The samples where run in quadruplicate on each plate (intraplate repeatability) and performed on three different days (interplate repeatability). Runs with a % CV above 15% are highlighted in bold.

		**Intraplate CV**				**Interplate CV**
**Serum**	**Antigen**	**Intraplate 1**	**Intraplate 2**	**Intraplate 3**	**Mean CV**	
Positive (serum 19)	P25	4,4	2,5	10,3	5,7	8,8
Positive (serum 20)		8,4	**15,5**	4,7	9,5	14,4
Doubtful (serum 21)		9,9	7,3	9,7	9,0	10,8
Negative (serum 22)		6,1	12,9	6,1	8,4	**23,93**
Negative (serum 23)		**17,5**	**20,4**	7	15,0	**26**
Positive (serum 19)	P16–25	2,5	1,4	9	4,3	7,1
Positive (serum 20)		6	13	5,7	8,2	**16,9**
Doubtful (serum 21)		7,9	9	11,6	9,5	**21,2**
Negative (serum 22)		6,1	**22,5**	4,4	11,0	**38,6**
Negative (serum 23)		12	**15,9**	12,7	13,5	**26**
Positive (serum 19)	SU5	3,6	1,2	2,8	2,5	5,2
Positive (serum 20)		3,7	5	0,5	3,1	6,1
Doubtful (serum 21)		9	6,6	5,8	7,1	10,2
Negative (serum 22)		5,6	14,9	7,3	9,3	**26,2**
Negative (serum 23)		14,2	**20,5**	14,3	**16,3**	**18,1**
Positive (serum 19)	TM-A	1,6	0,9	1,7	1,4	3,8
Positive (serum 20)		3	5	0,3	2,8	2,7
Doubtful (serum 21)		5,6	9,8	8,1	7,8	**17,7**
Negative (serum 22)		9,1	7,1	9	8,4	**21,8**
Negative (serum 23)		12,5	9,5	11,1	11,0	**20,7**
Positive (serum 19)	GST	5,7	1	6	4,2	5,8
Positive (serum 20)		8,5	9,6	3	7,0	4,9
Doubtful (serum 21)		8,5	2,8	9	6,8	12,6
Negative (serum 22)		8,1	10,7	6,6	8,5	3,7
Negative (serum 23)		13,8	10,2	7,2	10,4	**16,3**
Positive (serum 19)	BSA control	4,6	7,6	11	7,7	5,9
Positive (serum 20)		**19,6**	**16,2**	13,8	**16,5**	4,7
Doubtful (serum 21)		4,5	14,9	12	10,5	5
Negative (serum 22)		9,9	**23,5**	12,9	**15,4**	**17,1**
Negative (serum 23)		14,4	**30,9**	13,5	**19,6**	**24,6**
